# Dual effect of quercetin on rat isolated portal vein smooth muscle contractility

**Published:** 2010-06

**Authors:** Witness DH Chiwororo, John AO Ojewole

**Affiliations:** Department of Pharmacology, University of KwaZulu-Natal, Durban, South Africa; Department of Pharmacology, University of KwaZulu-Natal, Durban, South Africa

**Keywords:** rat isolated portal vein, quercetin, dual effects, endothelium-dependent relaxing factor, prostacyclin, cAMPdependent protein kinases

## Abstract

**Summary:**

This study examined the effects of quercetin on spontaneously contracting portal veins isolated from healthy young adult male and female Wistar rats (250–300 g). Quercetin (10^-7^–10^-4^ M) always produced significant biphasic effects, comprising an initial brief stimulant effect (rise in basal tone), followed by a sustained, longer-lasting secondary relaxant (inhibitory) effect on the venous tissues. The initial brief contractions of the venous muscle preparations were not modified by preincubation of the tissues with prazosin (10^-6^ M), suggesting that the initial upsurge in basal tone and increases in contractile frequencies of the venous tissues were probably not mediated via alpha_1_-adrenoceptor stimulation. However, preincubation of the tissues with nifedipine (10^-7^ M) significantly suppressed (*p* < 0.05) or attenuated the initial stimulant effect of quercetin, suggesting that the flavonoid might be activating L-type voltage-dependent calcium channels. The vasorelaxant effect of quercetin was partially but not significantly (*p* > 0.05) inhibited by L-NAME (100 μM) or indomethacin (10 μM), suggesting that the vasorelaxant effect of the flavonoid was unlikely to be mediated via endothelium-dependent relaxing factor (EDRF), or through prostacyclin (PGI_2_) pathways. N-p-tosyl-l-phenylalanine-chloromethyl-ketone (TPCK, 3 μM) significantly (*p* < 0.01) antagonised quercetin-induced relaxations, suggesting that cAMP-dependent protein kinases might have contributed, at least in part, towards the vasorelaxant effect of quercetin on rat isolated portal veins.

## Summary

Quercetin is a naturally occurring polyphenolic bioflavonoid that is widely distributed throughout the plant kingdom, including edible plants, mainly onions, apples, grapes, guavas, avocados, herbs, spices, berries and grains, and beverages, mostly teas and red wines.^1^ Several epidemiological studies have revealed that the Mediterranean diet, based primarily on dietary flavonoids (mainly quercetin) correlates with increased longevity,^2^ and decreased incidence of cardiovascular diseases.^3^-^6^

Quercetin has been shown to possess a wide spectrum of physiological and pharmacological properties responsible for its beneficial effects on the cardiovascular system.^7^ In fact, quecetin modifies eicosanoid biosynthesis, resulting in antiprostanoid and anti-inflammatory responses; protects low-density lipoprotein from oxidation, thus preventing atherosclerotic plaque formation; and prevents platelet aggregation and promotes relaxation of vascular smooth muscles.^1^,^8^ Studies by Duarte and co-workers have shown that quercetin possessed antihypertensive effects and lowered left ventricular hypertrophy, endothelial dysfunction and plasma and hepatic oxidative status in spontaneously hypertensive rats.^9^,^10^

The pharmacological effects of flavonoids on plasma membrane ion transport proteins such as Ca^2+^-Mg^2+^-ATPase, Na^+^-K^+^-ATPase and mitochondrial ATPase, as well as inhibition of cAMP- or cGMP-phosphodiesterase and protein kinases have been described.^11^-^15^ Moreover, the possible involvement of quercetin on L-type calcium channels has been reported.^1^,^16^ The possible involvement of myosin light-chain kinase in flavonoid-induced smooth muscle relaxation has also been raised.^17^-^19^ Quercetin has been reported to inhibit Ca^2+^-sensitising mechanisms in contractile proteins such as protein kinase C.^7^ Other earlier investigators have postulated a different mechanism which involves intracellular cAMP increase^11^-^14^ due to inhibition of cAMP-phosphodiesterase (PDE).^20^-^22^

Earlier studies have shown that quercetin and its metabolites (isorhamnetin, tamarixetin and kaempferol) exhibited vasodilatory effects on rat isolated aortic ring preparations.^18^,^23^,^24^ Generally, flavonoids have been postulated to evoke their vasodilatory effects through the release of endothelium-derived relaxing factors such as nitric oxide (NO) and prostacyclin (PGI2).^18^,^25^ However, there have been conflicting reports about the role of endothelium in the flavonoids’ vasorelaxant effects. Indeed, the vasorelaxant effects of several groups of flavonoids have been demonstrated to be endothelium dependent25 or endothelium independent.^26^,^27^

An extensive literature search has failed to reveal any study dealing with the effects of quercetin on portal vein preparations. The present study, therefore, examined the effects of quercetin on rat isolated portal veins, and attempted to characterise the possible underlying mechanisms involved in quercetin-induced vasorelaxation.

## Methods

Experimental protocols and procedures used in this study were approved by the Animal Ethics Committee of the University of KwaZulu-Natal and conform to the *Guide to the Care and Use of Laboratory Animals in Research and Teaching.*^28^

The pharmacological effects of quercetin were investigated on isolated, spontaneously contracting portal veins taken from naïve, normotensive, healthy young adult male and female Wistar rats (weighing 250–300 g). The animals were kept under conventional laboratory conditions of temperature, humidity and light, and allowed free access to food (standard pellet diet) and tap drinking water *ad libitum*. All the animals used were fasted for 16 hours, but still allowed free access to water prior to the commencement of our experiments. Each rat was euthanased by halothane inhalation, following which the abdomen was opened and the portal vein (with an *in situ* length of 2–3 cm) was quickly removed. The harvested venous muscles were cleaned free from fat and connective tissues and trimmed.

## Effect of quercetin on rat isolated portal veins

Each isolated portal vein segment was suspended under an applied resting tension of 0.5 g in a 30-ml Ugo Basile organ bath containing Krebs-Henseleit physiological solution (KHS) of composition, in mM: NaCl, 118; KCl, 4.7; NaHCO_3_, 25.0; MgCl_2_, 1.2; CaCl_2_.2H_2_O, 2.52; NaH_2_PO_4_.2H_2_O, 1.28; and glucose, 5.55; pH adjusted to 7.4. The bathing KHS was maintained at 35 ± 1^o^C and continuously aerated with carbogen (i.e. 95% O_2_ + 5% CO_2_ gas mixture). The mounted portal vein preparations were subsequently left to equilibrate for 45 to 60 minutes, during which time the bathing solution was changed every 15 minutes, before they were challenged with graded concentrations of quercetin (10^-7^–10^-4^ M) and/or reference drugs at different times.

Quercetin and/or the reference drug solutions were added to the bath fluid sequentially. They were repeated (where necessary) after washing out the previous quercetin or reference drug concentration four to five times, and allowing each tissue preparation to rest for five to 10 minutes, or until its tone returned to the control baseline level. In order to make allowance for changes in tissue sensitivity, two isolated portal veins were always set up at a time, one used as distilled water-treated control, and the other as quercetin- or reference drug-treated test preparation. The control venous muscle strips were only treated with volume/s of distilled water equivalent to the volume/s of quercetin or reference drug solutions.

To find out whether the initial brief contractile effect of quercetin on portal vein preparations was mediated through alpha_1_-adrenoceptor stimulation or L-type voltage-operated calcium channels, some of the portal vein preparations used were pretreated with an alpha1-adrenoceptor blocker, prazosin (10^-6^ M), or an L-type voltage-dependent calcium channel blocker, nifedipine (10^-7^ M), respectively, 20 minutes before the addition of quercetin to the bath fluid. The possible involvement of endothelium-derived relaxing factor (EDRF) and prostacyclin (PGI_2_) in quercetin-induced vasorelaxations was also investigated by pre-treating the venous tissues with N^G^-nitro-L-arginine methyl ester (L-NAME, 100 μM), a nitric oxide synthase inhibitor; or indomethacin (10 μM), a prostanoid synthase inhibitor, respectively, 20 minutes prior to addition of quercetin to the bath fluid.

Functional endothelium removal procedure was confirmed by lack of relaxant effect to a bolus of acetylcholine (10^-6^ M) administration. The possible contribution of cAMP-dependent protein kinases towards the relaxant effect of quercetin was examined by pre-incubating the venous tissues with N-p-tosyl-lphenylalanine-chloromethyl-ketone (TPCK, 3 μM), 20 minutes prior to addition of quercetin to the bath fluid. Quercetin- and/or reference drug-induced responses of the smooth muscle preparations were recorded isometrically by means of Ugo Basile force–displacement transducers and pen-writing two-channel Gemini recorders (model 7070).

## Drugs

Quercetin dihydrate, L-NAME, acetylcholine chloride, nifedipine hydrochloride, prazosin hydrochloride and indomethacin were purchased from Sigma-Aldrich Inc. (St Louis, MO, USA). TPCK was purchased from Bachem (Budendorf, Switzerland). The salts used to prepare Krebs-Henseleit physiological solution were purchased from Merck (Germany).

Except for quercetin and indomethacin, all drug solutions used were prepared by dissolving weighed amounts of the respective salts in distilled water. Quercetin was dissolved in dimethylsulfoxide (DMSO). The final concentration of DMSO was less than 0.08%, which was shown to be devoid of any observable pharmacological or physiological effect on the smooth muscle contractile tone. Indomethacin was dissolved in 0.5% w/v sodium bicarbonate immediately before use. Further dilutions of the drugs were made in KHS. Drug concentrations quoted in the text refer to final organ bath concentrations.

## Statistical analysis

All experimental data obtained are expressed as means (± SEM). Distilled water-induced control means were used as baseline values. The differences in responses among the different groups were analysed for statistical significance using the Student’s *t*-test and two-way analysis of variance (ANOVA, 95% confidence interval – GraphPad PRISM software, Version 5.00) followed by Dunnett’s *post-hoc* test. Multiple comparisons of the means were performed using Bonferroni’s test. In all cases, values of *p* ≤ 0.05 were taken to imply statistical significance.

## Results

The rat isolated portal veins used in this study always exhibited spontaneous, rhythmic, myogenic contractions with an average amplitude of 425 ± 13 mg (*n* = 8). Quercetin (10^-7^–10^-4^ M) raised the basal tone and caused concentration-dependent and significant reductions (*p* < 0.05–0.001) in the contractile amplitudes of the myogenic contractions. The inhibitory effects of quercetin on the contractile amplitudes of the veins were always preceded by initial brief but significant rises (*p* < 0.05) in the basal tones and, in 75% of the preparations set up, increases in contractile amplitudes, followed by more pronounced and longer-lasting secondary relaxations.

**Fig. 1.**
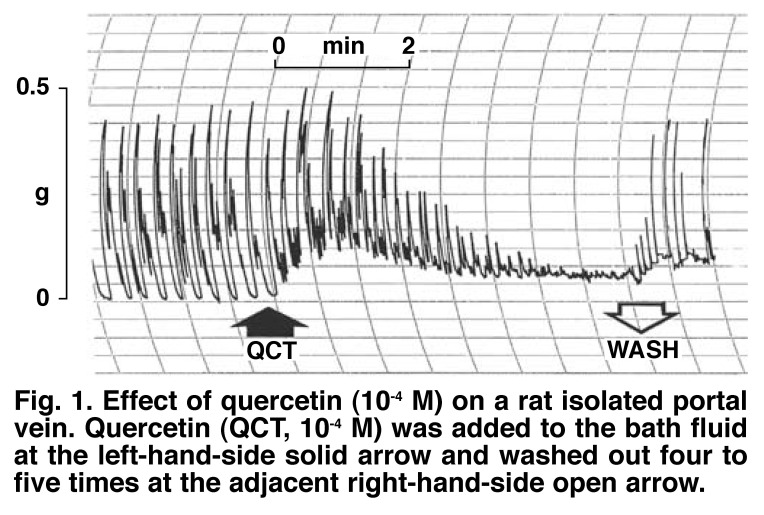
Effect of quercetin (10^-4^ M) on a rat isolated portal vein. Quercetin (QCT, 10^-4^ M) was added to the bath fluid at the left-hand-side solid arrow and washed out four to five times at the adjacent right-hand-side open arrow.

**Fig. 2.**
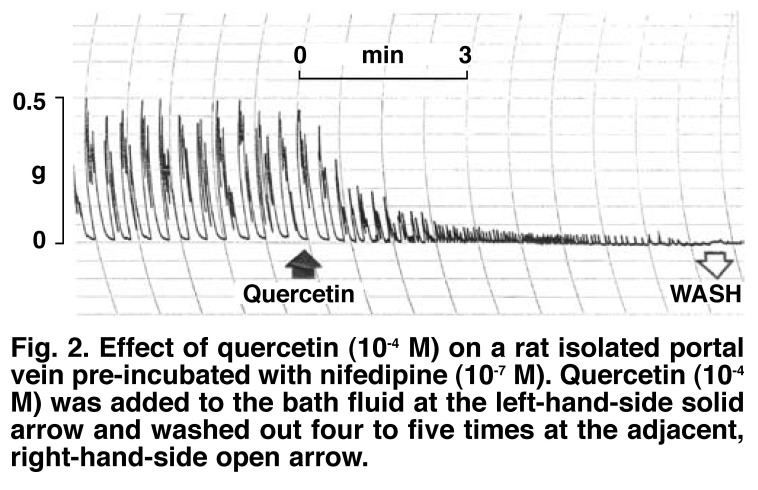
Effect of quercetin (10^-4^ M) on a rat isolated portal vein pre-incubated with nifedipine (10^-7^ M). Quercetin (10^-4^ M) was added to the bath fluid at the left-hand-side solid arrow and washed out four to five times at the adjacent, right-hand-side open arrow.

Fig. 1 shows a typical trace obtained with quercetin (10^-4^ M). The vasorelaxant effects of quercetin were completely or near completely reversed by washing out the quercetin concentrations four to five times, and subsequently allowing the venous tissues to rest for five to 10 minutes. Pre-incubation of the portal vein preparations with prazosin (10^-6^ M) did not modify the initial contractile effect of quercetin on the muscles, whereas preincubation of the tissues with nifedipine (10^-7^ M) significantly suppressed (*p* < 0.05) or attenuated the initial stimulant effects of quercetin, suggesting that quercetin might be activating L-type voltage-dependent calcium channels (Fig. 2).

**Fig. 3.**
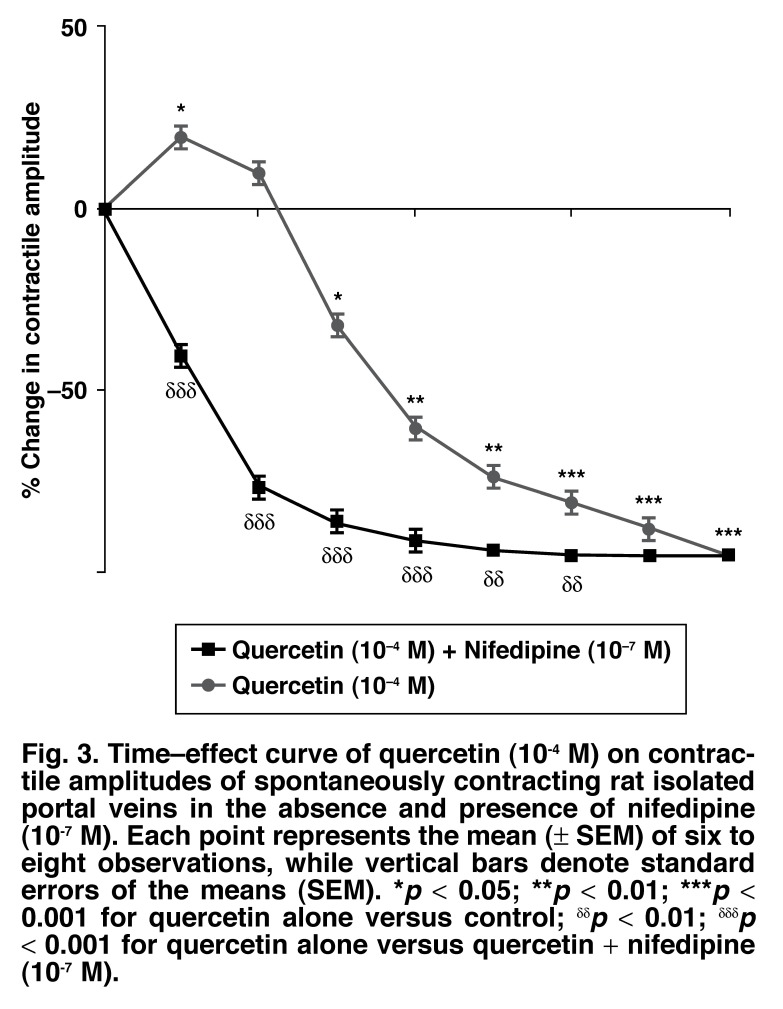
Time–effect curve of quercetin (10^-4^ M) on contractile amplitudes of spontaneously contracting rat isolated portal veins in the absence and presence of nifedipine (10^-7^ M). Each point represents the mean (± SEM) of six to eight observations, while vertical bars denote standard errors of the means (SEM). *p < 0.05; **p < 0.01; ***p < 0.001 for quercetin alone versus control; ^δδ^*p* < 0.01; ^δδδ^*p* < 0.001 for quercetin alone versus quercetin + nifedipine (10^-7^ M).

**Fig. 4.**
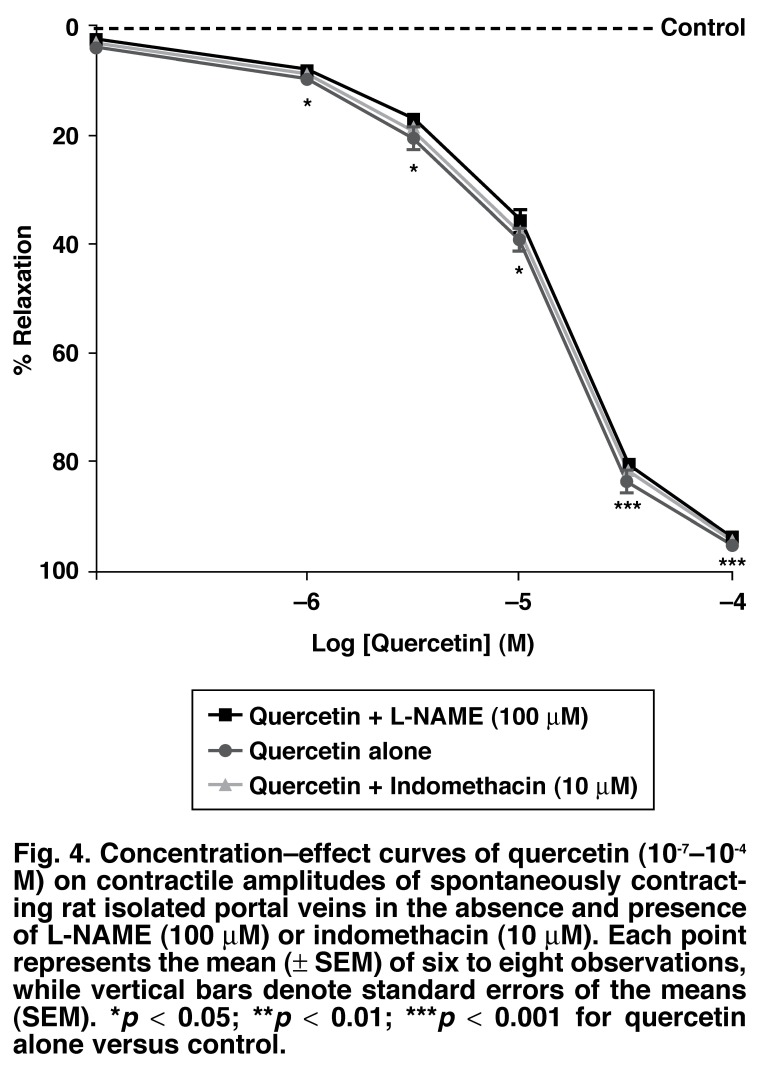
Concentration–effect curves of quercetin (10^-7^–10^-4^ M) on contractile amplitudes of spontaneously contracting rat isolated portal veins in the absence and presence of L-NAME (100 μM) or indomethacin (10 μM). Each point represents the mean (± SEM) of six to eight observations, while vertical bars denote standard errors of the means (SEM). *p < 0.05; **p < 0.01; ***p < 0.001 for quercetin alone versus control.

Fig. 3 summarises the time effect of quercetin (10^-4^ M) in the absence and presence of nifedipine (10^-7^ M). On their own, neither L-NAME (100 μM) nor indomethacin (10 μM) modified the contractile amplitudes of the spontaneously contracting preparations [425 ± 13 vs 420 ± 11 mg (L-NAME) and 427 ± 15 mg (indomethacin), respectively]. However, the vasorelaxant effects of quercetin were only slightly inhibited but not significantly (*p* > 0.05) by either nitric oxide synthase inhibitor, L-NAME (100 μM) or prostanoid synthase inhibitor, indomethacin (10 μM), suggesting that the vasorelaxant effect of quercetin was probably neither mediated via endothelium-dependent relaxing factor (EDRF), nor through prostacyclin (PGI_2_) pathways (Fig. 4).

**Fig. 5.**
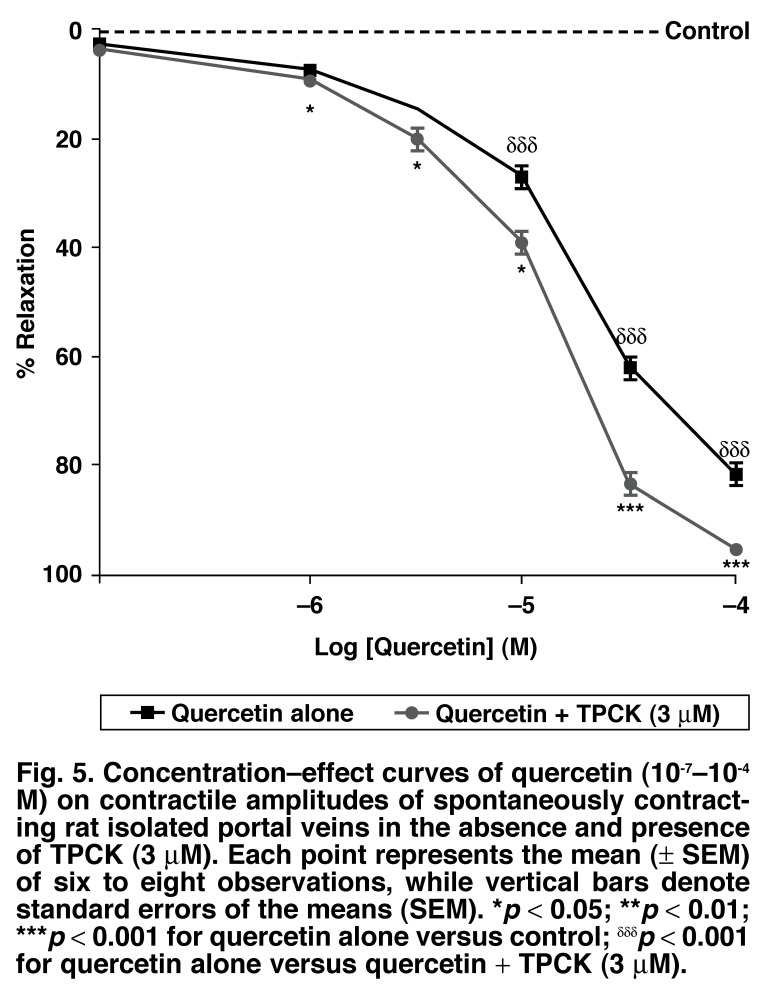
Concentration–effect curves of quercetin (10^-7^–10^-4^ M) on contractile amplitudes of spontaneously contracting rat isolated portal veins in the absence and presence of TPCK (3 μM). Each point represents the mean (± SEM) of six to eight observations, while vertical bars denote standard errors of the means (SEM). *p < 0.05; **p < 0.01; ***p < 0.001 for quercetin alone versus control; ^δδδ^*p* < 0.001 for quercetin alone versus quercetin + TPCK (3 μM).

TPCK alone did not modify the contractile amplitudes of the spontaneously contracting preparations (425 ± 13 vs 419 ± 10 mg). However, TPCK significantly antagonised (*p* < 0.01) but did not completely abolish quercetin-induced vasorelaxation, causing a shift of quercetin IC_50_ value from 12.9 ± 0.7 μM (quercetin alone) to 16.6 ± 0.8 μM (quercetin + TPCK) (Fig. 5).

## Discussion

Previous studies have shown that quercetin exhibited vasodilator effects in rat isolated aorta.^23^,^24^ The results of the present study indicate that the inhibitory effect of quercetin on portal vein preparations was usually preceded by initial brief but significant (*p* < 0.05) rises in the basal tone and, in 75% of the venous preparations set up, increases in contractile frequencies, followed by more pronounced and longer-lasting secondary relaxations of the venous tissues. However, the quercetin-provoked initial brief contractions of the muscle preparations were not modified by pre-incubation with prazosin (10^-6^ M). This observation suggests that the initial brief contractile effects of quercetin on the basal tones and increases in contractile frequencies of the preparations were unlikely to have been mediated via alpha_1_-adrenoceptor stimulation.

The possible role of Ca^2+^ influx in the quercetin-induced initial rise in the basal tone and increases in contractile frequencies of the preparations was examined by pre-incubating the venous tissues with nifedipine (10^-7^ M) before challenging them with quercetin. Nifedipine inhibits contractions of smooth muscles by reducing extracellular Ca^2+^ influx through a direct action on structural proteins of the L-type calcium channels.^29^ The partial blockade of the quercetin-induced initial brief contraction of the portal vein by nifedipine (10^-7^ M) probably suggests partial blockade of the influx of extracellular Ca^2+^ through L-type voltage-dependent calcium channels.^29^ This observation confirms and extends the earlier proposal that quercetin is a novel activator of L-type voltage-dependent calcium channels.^1^

The effect of quercetin is rather specific to L-type calcium channels, since T-type calcium channels were not affected by quercetin in an earlier study by Saponara *et al*.^01^ However, quercetin’s activation of L-type calcium channels would seem to contradict its well-known vasodilatory effect,^23^,^24^ as it would be expected to cause contraction of the vascular musculature. Therefore, the myorelaxant effect of quercetin on vascular tissue preparations originates from its reaction with a second target beyond the Ca^2+^ channel, which hierarchically prevails over the increase in Ca^2+^ influx expected from L-type calcium channel stimulation.^1^ It is, therefore, not unreasonable to speculate that the quercetin-induced initial contractile effects are likely to be as a result of a sudden influx of calcium into cells due to activation of L-type calcium channels by the flavonoid.

The possible involvement of endothelium mediators such as EDRF and PGI_2_ in quercetin-induced vasodilation was investigated by pre-treating the venous tissues with N^G^-nitro-L-arginine methyl ester (L-NAME, 100 μM), a nitric oxide synthase inhibitor, and indomethacin (10 μM) (to block prostanoid production), respectively, 20 minutes prior to addition of quercetin to the bath fluid. Pre-treatment of the portal vein tissues with either L-NAME or indomethacin did not significantly (*p* > 0.05) modify the vasorelaxant effects of quercetin, suggesting that quercetin-induced vasodilation is not mediated via EDRF, or through the PGI_2_ pathways.

The findings of this study are in agreement with, and extend the observations of, Duarte *et al*.^24^ and Pérez-Vizcaíno *et al*.,^19^ who noted that quercetin exhibited endothelium-independent vasodilation effects *in vitro*. However, in a chronic study by Duarte *et al*.^9^ quercetin restored endothelium-dependent relaxation, indicating that quercetin exerted marked acute vasodilator effects *in vitro* when administered intravenously. Chronic studies have also revealed that quercetin restored impaired endothelial function *in vitro*.^9^,^10^ Recent available data suggest that the vascular beneficial effects of flavonoids are closely related to their free-radical scavenging and anti-oxidant properties, which might thus protect NO from superoxide-induced inactivation.^30^,^31^ Quercetin is a potent anti-oxidant and has been shown to protect NO from scavenging actions of superoxide anions.^30^

The possible involvement of cAMP-dependent protein kinases on the relaxant effect of quercetin was examined by pre-incubating the venous tissues with TPCK (3 μM) 20 minutes prior to addition of quercetin. TPCK significantly (*p* < 0.01) antagonised but did not completely abolish quercetin-elicited vasorelaxation, suggesting that an intracellular upsurge in cAMP, due to cAMP-dependent protein kinase inhibition, might contribute, at least in part, towards the relaxant effects of quercetin on the spontaneously contracting portal veins. The mechanisms of the cAMP increase by quercetin could have been mediated via inhibition of cAMP phosphodiesterase.^11^-^13^,^26^,^28^ The results of the present study are also in agreement with those reported by Revuelta *et al*.^28^

Overall, the findings of the present study indicate that: (1) quercetin inhibited spontaneous contraction of, and relaxed rat isolated portal veins in a concentration-related manner; (2) quercetin is a novel activator of L-type voltage-dependent calcium channels; and (3) quercetin elicited an upsurge in intracellular cAMP, resulting in vascular smooth muscle relaxation. The myorelaxing properties of quercetin observed in this study lend pharmacological support to epidemiological studies, which postulate an inverse association between dietary flavonoid consumption and mortality from coronary heart diseases.
